# Effect of interferon on broilers’ fecal microbiome composition

**DOI:** 10.5455/javar.2025.l914

**Published:** 2025-05-16

**Authors:** Inna Burakova, Yuliya Smirnova, Mariya Gryaznova, Polina Morozova, Vyacheslav Kotarev, Ludmila Lyadova, Nadezhda Ivanova, Larisa Denisenko, Mikhail Syromyatnikov

**Affiliations:** 1 Laboratory of Metagenomics and Food Biotechnology, Voronezh State University of Engineering Technologies, Voronezh, Russia; 2 Department of Genetics, Cytology and Bioengineering, Voronezh State University, Voronezh, Russia; 3 All-Russian Veterinary Research Institute of Pathology, Pharmacology and Therapy, Voronezh, Russia

**Keywords:** Interferon, fecal microbiome, broiler, sequencing

## Abstract

**Objective:**

The purpose of our study was to investigate the effect of chicken interferon on the intestinal microbiota of broiler chickens.

**Materials and Methods:**

The study used next-generation sequencing on the Ion Torrent pragmatic general multicast platform to target the V3 *16S ribosomal ribonucleic acid* hypervariable region gene, allowing us to analyze in detail changes in the composition of the broiler chicken microbiome.

**Results:**

Forty-one bacterial genera were identified in the studied groups of broilers. The highest abundance in both groups was observed for *Lactobacillus*, which was 31.08% ± 6.52 in the control group and 36.08% ± 7.25 in the interferon group. There was no clustering between the microbiome communities of the groups studied. We found a decrease or complete absence of *Escherichia–Shigella, Eubacterium fissicatena group, Lachnospiraceae CHKCI001,* and *Pediococcus* in the interferon-treated broiler group compared to the control group and an increase in the number of genera *Ruminococcaceae CAG-352* and *Turicibacter* in the interferon group.

**Conclusion:**

A decrease in *E.–Shigella* may indicate normalization of the intestinal microbiota of broilers, and it can also be concluded that the introduction of interferon helps to suppress opportunistic bacteria. In the interferon group, a sharp increase in the number of *Turicibacter* was observed. Representatives of this genus are among the most common members in the intestines of broilers.

## Introduction

In the economics of the global market, poultry makes up a huge percentage of the protein source for the modern human diet and is also a common model animal for basic and applied research [1].

It is known that harmful environmental factors can shift the microbiome homeostasis of newborn chicks, which in turn leads to excessive growth of pathogens and the development of dysbiosis in broilers. Infectious diseases are also the main cause of mortality in chickens. The immune system plays an important role in protecting the body from infectious agents [2]. Thus, understanding the connection between microbiota and immune functions of organisms is one of the key tasks of modern research [3].

In the commercial breeding of broiler chickens, anti-microbial growth stimulants are widely used, as well as antibiotics in small doses to increase feed conversion efficiency [4]. However, over the years, many concerns have arisen about the overuse of antibiotics, leading to regulatory recommendations for their use. Thanks to this, several promising alternative drugs have emerged, which include modulation of the chicken gastrointestinal microbiome with prebiotics and probiotics, as well as antimicrobials [5,6].

It is known that interferon production is considered the most important innate immune response and the first line of defense against viral infections [7]. The antiviral activity of chicken interferon has been demonstrated in response to some infections caused by various viruses [8].

However, even though there is evidence that the intestinal microbiota can affect interferon production [9], there are no data on whether the introduction of interferon into the body can change the composition of the intestinal microbiota. In the therapy of farm animals, one of the main methods remains immunocorrection. The mechanisms of the influence of this therapy on the animal remain poorly studied. In addition, studies aimed at studying the effect of interferon therapy on the intestinal microbiota of chickens have not been carried out previously. Thus, the purpose of this work was to study the ways of action of interferons on the bacterial composition of the intestinal microbiota of broiler chickens at early stages of development. Thus, this study aimed to investigate the pathways of interferon action on the bacterial composition of the gut microbiota of broiler chickens at early stages of development.

## Materials and Methods

### Ethical Approval

Animal experiments were approved by the Ethical Commission of the All-Russian Veterinary Research Institute of Pathology, Pharmacology, and Therapy (VNIVIPFiT), Protocol No. 1-02/23, dated February 10, 2023. The experimental conditions complied with the requirements of the European Convention for the Protection of Vertebrates Used for Experiments or Other Scientific Purposes (ETS No. 123, Strasbourg, 1986).

### Study Plan

The object of the research was broiler chickens of the Cobb-500 cross. Broilers (24 birds) were divided into two equal groups, one of which was administered interferon. The birds of the experimental group were given the avian recombinant alpha interferon (structural and functional analog of endogenous interferon-alpha-2b) intranasally at a dose of 0.1 ml (1,000 IU/kg) per 1 kg of body weight, one day before vaccination (the age of the chickens was 13 days). Feces were collected after 30 days of interferon introduction under sterile conditions from the large intestine and immediately frozen at –20°С. The research was carried out based on the vivarium of the Federal State Budgetary Scientific Institution “All-Russian Research Veterinary Institute of Pathology, Pharmacology, and Therapy” within the framework of the state theme “Study the role of signaling cytokines in the pathogenesis of immunodeficiencies in birds and develop methodological approaches to the creation of means and methods of prevention.”

### Deoxyribonucleic Acid Extraction

Deoxyribonucleic acid (DNA) was isolated using the HiPure Microbiome DNA Kit (Magen, China). To 100 μg of a sample, 150 μl of Buffer DRB was added, mixed, and then incubated for 10 min. The 10 µl of DNase I was added, mixed, and then incubated for 20 min. Then, everything was transferred to new tubes with particles for resuspension. The 50 μl of Buffer ES was added and lysed. Centrifuge and transfer 400 μl of the supernatant into new tubes.

Add 400 µl of Buffer DL and 20 µl of Proteinase K. Vortex and incubate. The 400 μl of ethanol (96%) was added and mixed. Transfer 650 µl to a HiPure DNA Mini Column I placed in a collection tube, centrifuge, and discard the filtrate. Repeat the step twice. The 650 µl of Buffer GW1 was added, and the filtrate was centrifuged to remove it. We repeated this step with Buffer GW2. Place the column in a new 1.5 ml tube, add 50 µl Tris-EDTA buffer, incubate, and centrifuge.

### Sequencing

Sequencing was performed on the Ion Torrent pragmatic general multicast (PGM) platform. The V3 region of the bacterial *16S ribosomal ribonucleic acid* gene was chosen as the target segment of bacterial DNA. Bacterial DNA was isolated by real-time polymerase chain reaction (PCR) using universal primers: 337F (5’-GAC TCC TAC GGG AGG CWG CAG-3’); 518R (5’-GTA TTA CCG CGG CTG CTG G-3’). Amplified using 5X ScreenMix-HS (Evrogen, Russia) with the protocol: denaturation at 94°C for 4 min, 37 cycles: 94°C for 30 sec; 59°C for 30 sec; and 72°C for 30 sec, with final elongation at 72°C for 5 min. PCR products were purified with AMPure XP magnetic beads (Beckman Coulter, Life Sciences).

Further preparation of libraries was carried out with a commercial kit of NEBNext Fast DNA Library Prep reagents (New England Biolabs, USA). The protocol consists of the following steps: stripping the ends; connecting adapters; purification of finished libraries with AMPure XP magnetic particles (Beckman Coulter, Life Sciences); measurement of concentrations of purified libraries by quantitative PCR (qPCR) with a set of reagents Library Quantification Kit Ion Torrent Platforms (Kapa Biosystems, USA) with a protocol: primary denaturation at 95°C for 5 min, then 35 cycles: denaturation at 95°C for 30 sec; and elongation at 60°C for 45 sec. Analysis of melting curves 65–95 was also carried out, performing emulsion PCR using the OneTouch 2 device (Thermo Fisher Scientific, USA).

### Statistical Analysis

The Shannon index was used to calculate alpha diversity as well as to assess the overall representation and diversity of bacterial species in each study group. Alpha diversity differences were evaluated using the Wilcoxon rank sum test, adjusted by the false discovery rate method.

The microbiome’s intergroup similarity was evaluated through principal coordinate analysis using the Bray–Curtis difference. The statistical significance of the differences in the resulting distances was determined using permutational multivariate analysis of variance [10].

The MaAsLin2 package for R was used to conduct differential abundance analysis [11]. An adjusted *p* < 0.05 was considered statistically significant.

## Results

As a result of the sequencing performed on the Ion Torrent PGM platform, 41 bacterial genera were identified in the studied groups of broilers. The content of 25 bacterial genera was less than 1% for one of the study groups; they were grouped as “Others.” Figure 1 shows the top 16 bacterial genera for the study groups (Supplementary Table 1).

**Fig. 1.**
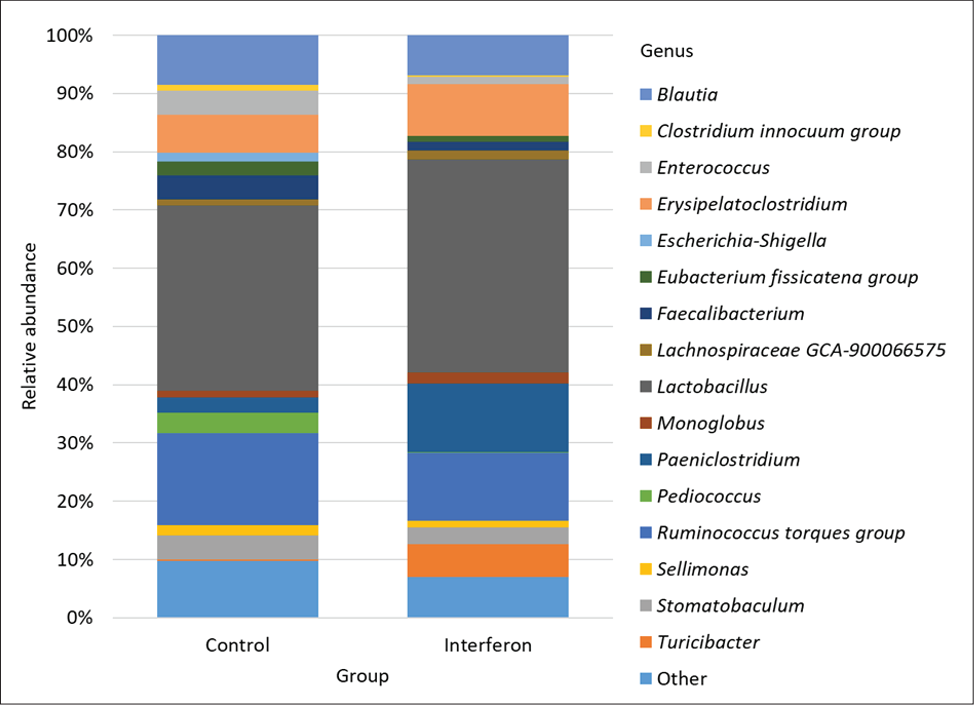
Figure 1. Bacterial composition of the study groups at the genus level.

**Table 1.** Supplementary Table 1. Relative abundance of top bacterial genera in control and interferon-treated groups.

Bacterial genus	Control (%)	Interferon (%)
Lactobacillus	31.08	36.08
Ruminococcus torques group	15.42	11.5
Blautia	8.25	6.83
Erysipelatoclostridium	6.33	8.83
Stomatobaculum	4.08	2.92
Faecalibacterium	4.08	1.58
Enterococcus	4.0	1.25
Pediococcus	3.42	<1
Paeniclostridium	2.5	11.67
Eubacterium fissicatena	2.33	<1
Sellimonas	1.67	1.08
Escherichia-Shigella	1.42	<1
Monoglobus	1.17	1.83
Clostridium innocuum	1.08	<1
Lachnospiraceae GCA-900066575	<1	1.42
Turicibacter	<1	5.5

The highest abundance in both groups was observed for *Lactobacillus*, which was 31.08% ± 6.52% in the control group and 36.08% ± 7.25% in the interferon group. The distribution of the other bacteria differed between the groups so that in the control group they were as follows: *Ruminococcus torque group* 15.42% ± 2.16%, *Blautia* 8.25% ± 1.52%, *Erysipelatoclostridium* 6.33% ± 1.02%, *Stomatobaculum* 4.08% ± 1.16%, *Faecalibacterium* 4.08% ± 2.48%, *Enterococcus* 4.0% ± 2.92%, *Pediococcus* 3.42% ± 2.70%, *Paeniclostridium* 2.50% ± 0.73%, *Eubacterium fissicatena group* 2.33% ± 0.67%, *Sellimonas* 1.67% ± 0.66%, *Escherichia–Shigella* 1.42% ± 0.70%, *Monoglobus* 1.17% ± 0.67%, and *Clostridium innocuum group* 1.08% ± 0.73%. The frequency of *Lachnospiraceae GCA-900066575* and *Turicibacter* was less than 1% in the control group. In the interferon group, the following distribution was observed: *Paeniclostridium* 11.67% ± 4.51%, *R. torques group* 11.50% ± 0.93%, *Erysipelatoclostridium* 8.83% ± 1.96%, *Blautia* 6.83% ± 0.89%, *Turicibacter* 5.50% ± 2.40%, *Stomatobaculum* 2.92% ± 0.62%, *Monoglobus* 1.83% ± 0.71%, *Faecalibacterium* 1.58% ± 0.45%, *Lachnospiraceae* GCA-900066575 1.42% ± 0.38%, *Enterococcus* 1.25% ± 0.72%, and *Sellimonas* 1.08% ± 0.23%. The abundance of *E. fissicatena* group, *C. innocuum group, Pediococcus*, and *E.–Shigella* was less than 1% (Supplementary Table 2; Supplementary Fig. 1).

**Table 2.** Supplementary Table 2. Statistically significant changes in bacterial genera abundance.

Bacterial genus	Control (%)	Interferon (%)	p-value
Escherichia-Shigella	1.42	0.0	0.02
Eubacterium fissicatena group	2.33	0.92	0.02
Lachnospiraceae CHKCI001	0.92	0.17	0.01
Pediococcus	3.42	0.08	0.04
Ruminococcaceae CAG-352	0.0	0.58	0.02
Turicibacter	0.25	5.5	0.02

**Fig. 1.**
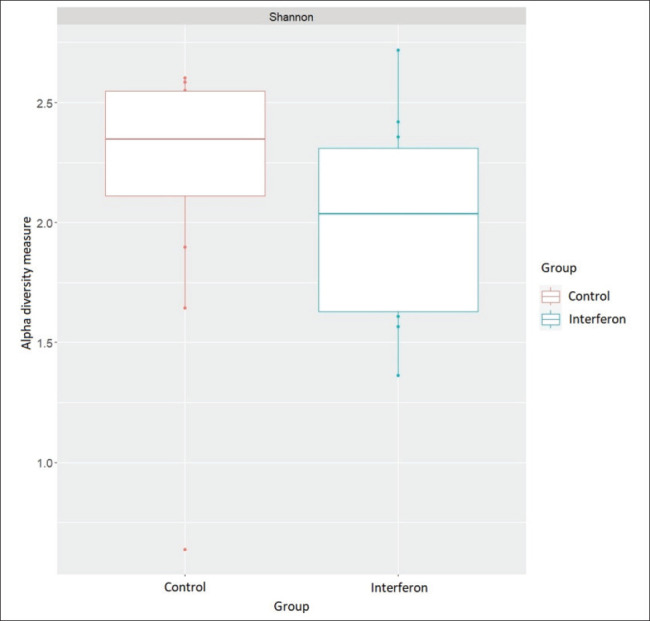
Supplementary Figure 1. Heatmap of differential abundance in key genera.

Figure 2 shows the alpha diversity scores for the study groups. The Shannon index, which describes the alpha diversity of the microbiome, was 2.16 ± 0.16 for the control group and 1.99 ± 0.12 for the interferon group (Supplementary Table 3; Supplementary Fig. 2). Thus, the microbiome of the control group was richer than that of the interferon group, but this difference was not statistically significant (*p* = 0.16). Figure 3 shows the beta diversity scores for the study groups. Figure 3 shows that there was no clustering between the microbiome communities of the groups studied (*p* = 0.45).

**Fig. 2.**
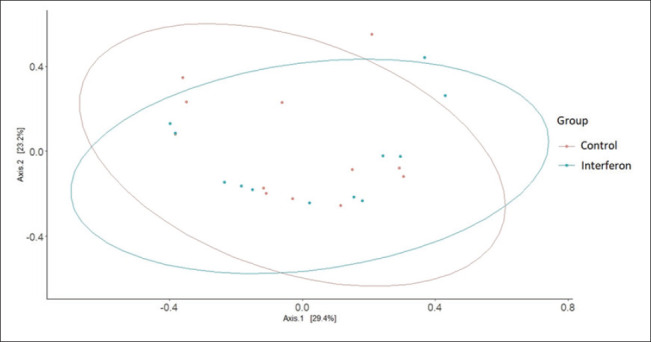
Figure 2. Indicator of intragroup diversity of the microbiome of the study groups.

**Fig. 3.**
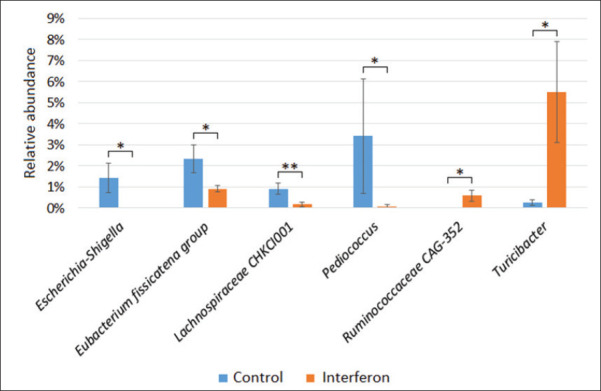
Figure 3. Indicators of intergroup microbiome diversity of the study groups.

**Fig. 2.**
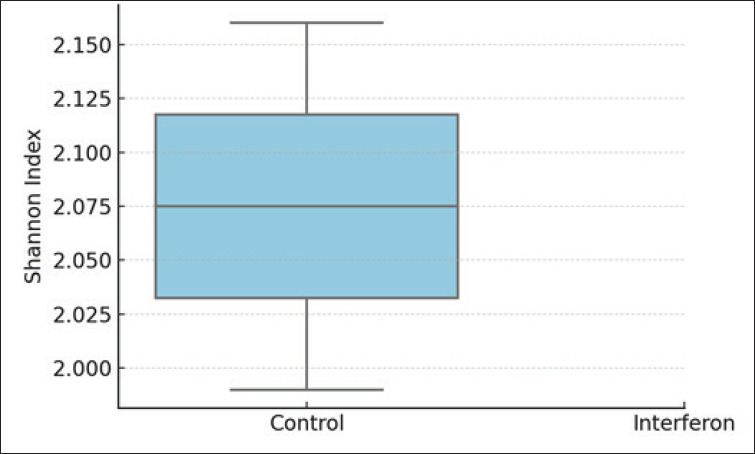
Supplementary Figure 2. Alpha diversity (Shannon Index) between groups.

**Table 3.** Supplementary Table 3. Alpha diversity index (Shannon index) between groups.

Group	Shannon index (Mean ± SD)	p-value
Control	2.16 ± 0.16	0.16
Interferon	1.99 ± 0.12	ns

**Fig. 4.**
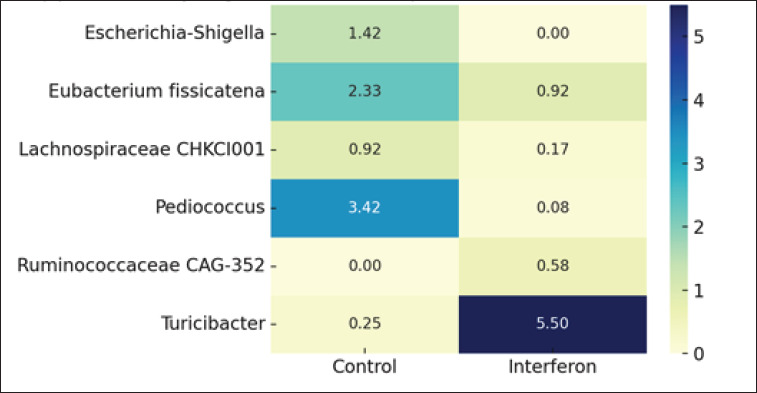
Figure 4. Differences in the abundance of bacterial genera between the study groups.

Differential abundance analysis showed the presence of statistically significant differences between the studied groups for six bacteria (Fig. 4).

We found a decrease or complete absence of *E.–Shigella* (0% *vs.* 1.42% ± 0.70, *p* = 0.02), *E. fissicatena group* (0.92% ± 0.15 *vs*. 2.33% ± 0.67, *p* = 0.02), *Lachnospiraceae CHKCI001* (0.17% ± 0.11 *vs*. 0.92% ± 0.26, *p* = 0.01), and *Pediococcus* (0.08% ± 0.07 *vs*. 3.42% ± 2.70, *p* = 0.04) in the interfer-on-treated broiler group compared to the control group and an increase in the number of genera *Ruminococcaceae CAG-352* (0.58% ± 0.26 *vs*. 0%, *p* = 0.02) and *Turicibacter* (5.50% ± 2.40 *vs*. 0.25% ± 0.13) in the interferon group, p = 0.02).

## Discussion

This research aimed to study the effect of interferon on the gut microbiome of broiler chickens. Even though treatment with immunomodulatory substances is the most widely used in agricultural animal husbandry, there are no studies aimed at studying the mechanisms of the effect of species-specific interferons on the gut microbiota of chickens or any other farm animals. However, it is known that intestinal bacteria and their metabolites can influence interferon signaling pathways [12]. This empha-sizes the high significance of our study, demonstrating a link between interferon treatment and bacterial changes in the gut of chickens. We have shown an indirect positive role of interferons on the gut of animals, which in turn may have a positive effect on the health of birds. In addition, we found bacterial taxa (*E.–Shigella*, *E. fissicatena* group, *Lachnospiraceae*, *Pediococcus Ruminococcaceae CAG-352*, and *Turicibacter*) that colonized the intestines of interfer-on-treated chickens to varying degrees.

The abundance of *E.–Shigella* decreases relative to the control in the interferon group of broiler intestinal micro-biota. Previous studies have indicated that *E.–Shigella* was one of the dominant genera of the bacterial community in the cecum of newly hatched chicks, but their abundance decreases with age [13]. It has also been demonstrated that *E.–Shigella* is a group of opportunistic bacteria that can destroy the intestinal structure and thereby exert pro-inflammatory activity through the production of virulence factors [14].

It was revealed that this bacterial species negatively correlates with growth as well as with fat digestibility in broilers. In turn, the addition of moderate antibiotics and additives with organic acids can suppress the number of *E.–Shigella* [15]. Again, it was found that vaccinated birds had a lower relative abundance of the bacterial genus *E.–Shigella*, which can negatively impact chicken health and produce foodborne illnesses [16]. A review of the literature data showed that a decrease in this bacterial genus may indicate normalization of the intestinal microbiota of broilers, and it can also be concluded that the addition of interferon helps to suppress opportunistic bacteria.

Bacteria of the *E. fissicatena* group are capable of producing butyrate, which not only maintains normal intestinal permeability but also has an anti-inflammatory effect [17]. This genus has been shown to play a protective role in the pathogenesis of endocarditis [18].

In turn, the *E. fissicatena group* exhibits a specific response to a high-fat diet, demonstrating a strong association with host obesity and associated metabolic disorders [19]. According to the results of our study, it was shown that the number of bacteria of the genus *E. fissicatena group* decreased in the interferon group relative to the control.

It is known that an increase in the abundance of *Lachnospiraceae CHKCI001* bacteria, representatives of the *Lachnospiraceae* family, producing SCFA, can maintain homeostasis of the intestinal microbiota and also affect the growth of broilers [20,21]. In several studies, an increase in this bacterium was observed in chickens kept on a multi-enzyme diet with the addition of wheat-soy flour or in combination with inactivated lactobacilli [22], as well as with the inclusion of antibacterial peptides in the diet [23]. Despite the literature data, according to the results of our study, it was found that the introduction of interferon inhibited the growth of *Lachnospiraceae CHKCI001*.

Similar results were also found for *Pediococcus* sp. which is considered a beneficial genus for the organism. According to the results of the research work by Hamid et al. [24], it was noted that broiler chickens fed with feed supplemented with *Pediococcus* had a reduced abundance of *E. coli* in the ileum compared to the control [24]. Additionally, Lee et al. [25] reported that supplements containing *Pediococcus* can have a negative effect on the growth of pathogenic bacteria and thus protect birds [25]. Thus, it is advisable to delve into the study of the inhibitory effect of interferon on the abundance of *Pediococcus* in the intestinal microbiome of broilers to confirm and explain this effect on the organism.

It was noted that *Ruminococcaceae CAG-352* bacteria densely populated the microbiome of laying hens with a low response to vaccination against infectious bronchitis virus [26]. The data of our study also demonstrated an increase in the abundance of the genus *Ruminococcaceae CAG-352* in the interferon group. It was previously established that the bacterial species *Turicibacter* is one of the most numerous in the intestinal microbiota of broilers [27]. However, this genus has been repeatedly noted as pathogenic and has also been associated with various pathological conditions in the body [28]. This is confirmed in the work of Song et al. [29], where an increase in this genus was noted after antibiotic therapy in broiler chickens [29]. Perhaps interferon has a similar effect on *Turicibacter* bacteria because, according to the results of our study, an increase in the abundance was noted in the interferon group relative to the control. Therefore, the role of *Turicibacter* in the broiler microbiome remains unclear.

We can assume that interferon indirectly affects the body by influencing the number of phages capable of infecting various bacterial taxa. The ability of interferons to regu-late the intestinal virome has been previously reported.

There are two main limitations in our study that could be addressed in the future. First, the study focused on studying only the bacterial composition; for a more complete characterization of the changes, it is necessary to add histological methods, as well as virome studies. Second, the limitation is the sample size of the study groups. To more accurately assess the effect of interferon on the intestinal microbiome of chickens, studies on larger groups from different regions are needed.

## Conclusion

It was revealed that the introduction of interferon intranasally can change the composition of the microbiota of the intestine of broilers. *Lactobacillus* bacteria were the most abundant in the large intestine of broilers in both groups studied. We found a decrease or complete absence of *E.–Shigella, E. fissicatena* group, *Lachnospiraceae CHKCI001,* and *Pediococcus* in the interferon-treated broiler group compared to the control group and an increase in the number of genera *Ruminococcaceae CAG-352* and *Turicibacter* in the interferon group.

## List of abbreviations

DNA, deoxyribonucleic acid

PCR, polymerase chain reac-tion

PGM, pragmatic general multicast.
